# Multiresidue Method for Analysis of β Agonists in Swine Urine by Enzyme Linked Receptor Assay Based on β_2_ Adrenergic Receptor Expressed in HEK293 Cells

**DOI:** 10.1371/journal.pone.0139176

**Published:** 2015-09-30

**Authors:** Jian Wang, Yongxin She, Miao Wang, Maojun Jin, Yongfei Li, Jing Wang, Yuan Liu

**Affiliations:** 1 Institute of Quality Standards and Testing Technology for Agro-products of CAAS, Key Laboratory of Agro-Product Quality and Safety, Ministry of Agriculture, Beijing, 100081, P. R. China; 2 Department of Food Science, Hebei North University, Zhangjiakou, 075000, P. R. China; Duke University, UNITED STATES

## Abstract

A novel enzyme-linked receptor assay (ELRA) based on β_2_-adrenergic receptor (β_2_-AR) has been developed for rapid and high-throughput detection of β-adrenergic agonists (β-agonists) in urine. Human embryonic kidney cells (HEK293) were introduced as the expression system to enhance the functionality of the recombinant β_2_-AR, and the attempt to detect β-agonists in swine urine using such approaches was accomplished unprecedentedly. In this article, a recombinant porcine β_2_-AR was produced in the inner membrane of HEK293 cells and purified from crude membrane protein by nickel-nitrilotriacetic acid affinity chromatography. After activity identification, the recombinant receptor was used in the development of direct competitive ELRA. Several parameters such as blocking buffer and blocking process were optimized and the performance of the system was determined. The IC50 concentrations of clenbuterol, salbutamol, and ractopamine were 34, 53 and 63 μg/L, and the average recovery rates were 68.2%, 60.3% and 65.5%, respectively. ELRA based on β_2_-AR shows a series of advantages such as safety, easy operation, and high efficiency, making it promising for the rapid screening of β-agonists in animal urine.

## Introduction

β-adrenergic agonists (β-agonists) were initially used to treat asthma and bronchial diseases in humans and animals. Later, these compounds were also found to be efficient repartitioning agents capable of improving muscular mass, inhibiting fat synthesis, and reducing the fat deposition in carcasses at a dose 10 times that of the clinical dosage [1–3]. However, the residues of β–agonists that accumulate in animal tissues could lead to cardiovascular and central nervous system effects in humans, including muscle tremors, palpitations, tachycardia, and dizziness [4]. Therefore, the administration of all β–agonists as growth promoters in livestock industry has been strictly banned in China [5] and the European Union [6]. Nevertheless, owing to the enormous economic benefits, the illegal abuse of such agents never stopped, which caused many incidents of poisoning. Furthermore, in addition to the abuse of some known β-agonists such as clenbuterol (CBL) and salbutamol (SAL), a series of novel β-agonist derivatives with similar structure and function have also been synthetized to evade detection by routine screening methods [7–8]. Thus, it is urgently needed to establish a high-throughput screening approach for multiresidue determination of β-agonists.

Till date, the commonly used analytical methods of β-agonists are based on chromatographic techniques and immunoassays. There are various chromatographic methods developed for the confirmation of β-agonists, such as ultra-performance liquid chromatography tandem mass spectrometry [9], gas chromatography–mass spectrometry [10], high-performance liquid chromatography [11], and capillary electrophoresis [12]. Although these techniques are greatly sensitive and accurate, they are unsuitable for field analysis and rapid screening, as they require expensive and sophisticated instruments and complicated and time-consuming sample pretreatment. In recent years, immunoassay methods represented by enzyme-linked immunosorbent assay and colloidal gold immunochromatographic assay have been commercially available [13–14]. In addition, some new screening methods such as surface plasmon resonance [15], electrochemical methods [16], surface-enhanced Raman scattering immunoassay [17], and fluorescence [18] have also been established. However, despite the high sensitivity and ideal specificity, they suffer from several disadvantages. A primary drawback is the tedious antibody preparation procedure so that only a small range of β-agonists can be detected [19–20]. Therefore, it is very difficult to detect multiresidues and perform unknown material analysis of β-agonists by the antibody-based immunoassay methods.

The receptor assay based on recombinant β_2_-adrenergic receptor (β_2_-AR) is an emerging and powerful alternative screening technique capable of detecting a wide spectrum of similar compounds, including new molecules without any detailed information and low-level cocktails of compounds. β_2_-AR is a member of the large superfamily of G-protein-coupled receptors, which can be activated by adrenaline and synthetic β-agonists [21]. The sites of interactions between agonists and the receptor [22] and the agonist-induced conformational switches [23–24] have been studied by mutagenesis and biophysical methods. At present, heterologous expression is the primary means of obtaining receptors due to the low availability and difficulty in separating and purifying natural receptors from animal cell membranes. The recombinant receptors could be used as biorecognition elements to detect β-agonists due to their continuous source and high affinity. The recombinant expression of functional β_2_-AR has been achieved in all possible expression systems, including *Escherichia coli* [25–26], yeast [27], insects [28], mammalian cells [29–30], and cell-free systems [31–32]. However, obtaining abundant and high-affinity recombinant protein for its practical application remains the toughest bottleneck. Currently, the receptor protein expressed in the mammalian cells is the closest approximation of the native receptor in structure, which has good application prospect [33].

At present, few of the radio-receptor assays have already been developed for multiresidue detection of β-agonists using natural membrane-bound β_2_-AR prepared from bovine teat muscles [34], or recombinant β_2_-AR expressed in Chinese hamster ovary cells [35], *E*. *coli* [36], and NCB20-D1 cells [37]. However, this form of analysis has its limitation in application owing to the high cost and the harmful effects of radioactive isotopes. Hence, it is a trend to develop nonradioactive multiresidue detection of β-agonists based on the recombinant β_2_-AR. The only related research was the one by G. Cheng et al [38], who developed an enzyme-linked receptor assay (ELRA) based on β_2_-AR expressed in Sf 9 insect cells for the detection of β-agonists. In general, the structure of protein expressed in mammalian cell is similar to that of natural protein when compared with insect cell, which leads to the same performance in activity. To increase the affinity of the core component (receptor), the cell line of the most common mammalian cell, human embryonic kidney cells (HEK293), was used to express β_2_-AR *in vitro*. Moreover, the established ELRA was the first attempt to detect β–agonists in swine urine, laying the foundation for practical application and commercialization.

## Materials and Methods

### Materials and reagents

SV Total RNA Isolation System, Access reverse transcription polymerase chain reaction (RT-PCR) System, and T4 DNA ligase were purchased from Promega (Madison, WI, USA). The restriction enzymes of NcoI and XhoI were purchased from NEB (Ipswich, MA, USA). Competent cell NovaBlue, pTriEx-1.1 Hygro vector, and GeneJuice® transfection reagent were supplied by Novagen (Billerica, MA, USA). Nickel–nitrilotriacetic acid (Ni–NTA) His Bindresin was purchased from Qiagen (Hilden, Germany). HEK293 and horseradish peroxidase (HRP)-β-agonists were supplied by Beijing Kwinbon Biotechnology Co., Ltd (Beijing, China). Dulbecco’s modified Eagle’s medium (DMEM) and fetal bovine serum (FBS) were obtained from Gibco (Grand Island, NY, USA). Anti-His monoclonal antibody and HRP-conjugated goat anti-mouse IgG were obtained from Beijing ComWin Biotech Co., Ltd (Beijing, China).

CBL, SAL, and ractopamine (RAC) were purchased from Sigma-Aldrich (St. Louis, MO, USA). HRP-β-agonists were gifts from Beijing Kwinbon Biotechnology Co., Ltd (Beijing, China). All chemicals were of analytical grade without any further purification.

### Urine and liver samples

Liver samples weighing 300 g were routinely collected from the slaughtered pig from a local meat processing factory. Urine samples were obtained from the pigs born into a strictly controlled livestock farm belonging to the China National Cereals, Oils, and Foodstuffs Corporation. They had been proved to be free of any β_2_ agonist by API 5000 LC–MS/MS (AB SCIEX) according to the method of Chinese Ministry of Agriculture announcement NO. 1063. Then the supernatant was obtained after centrifugation at 10,000 rpm for 30 minutes, which was used to measure the recovery rates of β-agonists.

### Gene cloning and plasmid construction

About 30 mg fresh liver tissues were prepared for total RNA extraction using the Qiagen SV Total RNA Isolation System as per the manufacturer’s instructions. The quality of the isolated RNA was controlled by agarose gel electrophoresis, and concentration and purity were determined by ultraviolet spectrophotometer. The cDNA of porcine β_2_-AR was amplified by RT-PCR, and the digestion sites of NcoI and XhoI were arranged at 5′ and 3′ ends of it. Primers were 5′-CATGCCATGGCAGGGCAGCCCGGGAACCGC-3′ (forward) and 5′-CCGCTCGAGTCACAGCATGGAGTCATTTGTACTACAGTTCCTCC-3′ (reverse) designed and synthesized on the basis of the published pig β_2_-AR gene sequence in Genebank (AF000134). The 1257-bp product that encoded 418 amino acids was inserted into pMD-18T vector to form the cloning plasmid pMD18-T-β_2_-AR. Recombinant expression plasmid of pTriEx-1.1 Hygro-β_2_-AR was constructed by restriction digestion and ligation with a C-terminal 6-His fusion tag. The plasmid can be used to test expression in *E*. *coli*, insect, and mammalian cells, as it contains the promoters of the above-mentioned 3 expression systems.

### Expression of β_2_-AR

The recombinant plasmid of pTriEx-1.1 Hygro-β_2_-AR was transformed into NovaBlue competent cells incapable of “blue/white” screening of recombinants by lacZ α complementation. Thus, selection of transformants was accomplished by plating on medium containing 50 μg/mL ampicillin. The recombinant plasmid was isolated from positive clones and then transiently transfected into HEK293 cells by GeneJuice transfection reagent after verification. The day before transfection, 4-5 × 10^6^ cells were plated in complete growth medium (DMEM + 10% FBS) per 10 cm dish and incubated at 37°C (5% CO_2_) overnight. When the cell density reached 50-80% confluence, 1 mL of GeneJuice transfection reagent/plasmid mixture (10 μg DNA: 30 μL reagent/mL of DMEM medium) was added dropwise to cells and incubated at 37°C for 4 hours. Subsequently, the medium was replaced with complete growth medium and the incubation time (48 hours, 72 hours, 96 hours) was optimized for protein expression.

### Crude membrane preparation

The membrane fraction was isolated from infected HEK293 cells as described earlier [39–40]. Four dishes of transfected cells were rinsed twice with ice-cold phosphate-buffered saline (PBS) and harvested in 7 mL of hypotonic lysing buffer (10 mM Tris-hydrochloride [HCl], pH 7.4) containing protease inhibitors (1 mM ethylenediaminetetraacetic acid, 10 μg/mL benzamidine, 10 μg/mL leupeptin, 20 μg/mL soybean trypsin inhibitor, 5 μg/mL aprotinin, and 0.2 mM phenylmethylsulfonyl fluoride). All further steps were carried out at 4°C. Then, the cell suspension was poured into a tissue homogenizer, and the cells were homogenized with 30 strokes. The suspension was centrifuged at 1500 rotations per minute (rpm) for 10 minutes, and the pellet was discarded. The supernatant fraction was centrifuged at 48,000*g* for 20 minutes. The membrane pellet was suspended in 7 mL of incubation buffer (50 mM Tris-HCl and 5 mM MgCl_2_, containing protease inhibitors as in hypotonic lysing buffer) and centrifuged again as mentioned above. The final pellets were suspended in 1 mL of the above-mentioned buffer and aliquots of the suspension were frozen at −80°C or in liquid nitrogen.

### Purification of recombinant β_2_-AR

Cell pellets were resuspended in10-fold volume of hypotonic lysing buffer, and the suspension was homogenized using a dounce homogenizer (20 strokes). The suspension was centrifuged at 48,000*g* for 30 minutes, after which the pellets were resuspended in hypotonic lysing buffer, and the suspension was centrifuged at 48,000*g* for 30 minutes again. After weighing, the membrane pellet was resuspended in solubilization buffer (20 mM Tris-HCl, pH 7.4 containing 500 mM NaCl, 10% glycerol, 1% *n-*dodecyl-b-D-maltoside, and the protease inhibitors as in hypotonic lysing buffer) using a homogenizer (30 strokes), and the suspension was mixed by nutation at 4°C for 1 hour. Then the solution was centrifuged at 48,000*g* for 30 minutes to remove any insoluble particulate material. Subsequent purification of β_2_-AR by Ni-NTA-affinity chromatography was carried out as per the manufacturer’s protocol. The concentration of midazolein elution buffer was optimized between 75 mM and 250 mM. The fractions were analyzed by sodium dodecyl sulfate polyacrylamide gel electrophoresis (SDS-PAGE) and Western blot (Anti-His monoclonal antibody as the first antibody and goat-anti-mouse antibody as the second antibody) and their protein concentration was determined by bicinchoninic acid assay method. The final receptor was stored at −80°C after it was frozen in liquid nitrogen.

### Activity assay and ELRA measurements

The principle of competitive ELRA was similar to competitive ELISA, which was described earlier [39]. In brief, 100 μL of diluted receptor solution was added to each microwell and the plate was incubated at 4°C overnight. After the wells were emptied completely and washed once with 0.2% PBS-T (PBS containing 0.2% Tween 20), excess binding sites were blocked by 1% bovine serum albumin (BSA) at 37°C for 2 hours. After washing the microplate thrice, 50 μL of standard solution (0, 10, 50, 100, 500, 1000 μg/L) or sample was added to each well and co-incubated with 50 μL of HRP-β-agonist solution at 37°C for 30 minutes. A quantity of 100 μL of substrate/chromogen (TMB) solution was added to each well and incubated at 37°C for 15 minutes. The reaction was terminated by adding 50 μL of stop reagent (2 M sulfuric acid) and the absorbance at 450 nm was measured on Multiskan MK3 microplate reader (Thermo Fisher, Waltham, MA, USA).

### Method validation

The inhibition concentrations (IC50 values) for inhibition of binding to the receptor by β-agonist were calculated from standard curves fitted by logarithm model, which plotted the β-agonist concentration versus the percentage of binding (B/B_0_). The method of recovery was determined at 4 different levels using urine samples spiked with 3 β-agonists (CBL, RAC, and SAL) at standards of 1, 10, 50, and 100 μg/L (5 replicates per concentration level), respectively. In trial test, we had tested 5 β-agonists including clenbuterol, salbutamol, ractopamine, carbuterol and terbutaline. The results showed that the aniline-type β-agonists clenbuterol had the highest affinity, next were the phenolic-type β-agonists, such as ractopamine and salbutamol, and yet carbuterol and terbutaline showed the lowest affinity. On the basis of the above study, we chose the 3 β-agonists of clenbuterol, salbutamol and ractopamine with higher affinity as the detection targets. Before adding standards, blank urine samples should be centrifuged for 10 minutes (5000 rpm) to obtain the supernatant.

To describe the smallest concentration that can be reliably measured by the analytical procedure, the limit of detection (LOD) was calculated as the mean + 3 standard deviations (SDs) based on 20 blank samples. For the assessment of precision on calibration graphs, coefficients of variation (CV) were determined by the analysis of the spiked CBL at 4 levels for 5 different analyses.

### Data analysis

Data reported were mean ± SD. Statistical analysis of the data and non-linear curve fitting were performed using Microsoft Excel 2010 and Origin 8.5, respectively.

## Results and Discussion

### Preparation of the recombinant β_2_-AR

To obtain abundant active β_2_-ARs is the premise for the development of receptor assay used for the multianalyte detection of β-agonists. In this article, there was increased production of porcine β_2_-AR in the inner membrane of HEK293 cells, which successfully retained its binding affinity after purification. The full-length cDNA of β_2_-AR was 1257 bp and was submitted to Genbank under the accession number KF023571.1. Sequencing revealed 4 mutations (85G→A, 1056T→C, 1155G→C, and 1156C→G) in the gene compared with the published gene sequence (GenBank accession number AF000134). The mutation resulted in 3 amino acid changes (29Asp→Asn, 385Asp→Glu, 386Ala→Pro), which might be attributed to gene diversity of β_2_-AR in different porcine breeds. Furthermore, all of the amino acids at the ligand binding sites were cloned correctly so as to lay a foundation for ensuring the function of the recombinant receptor.

**Fig 1:**
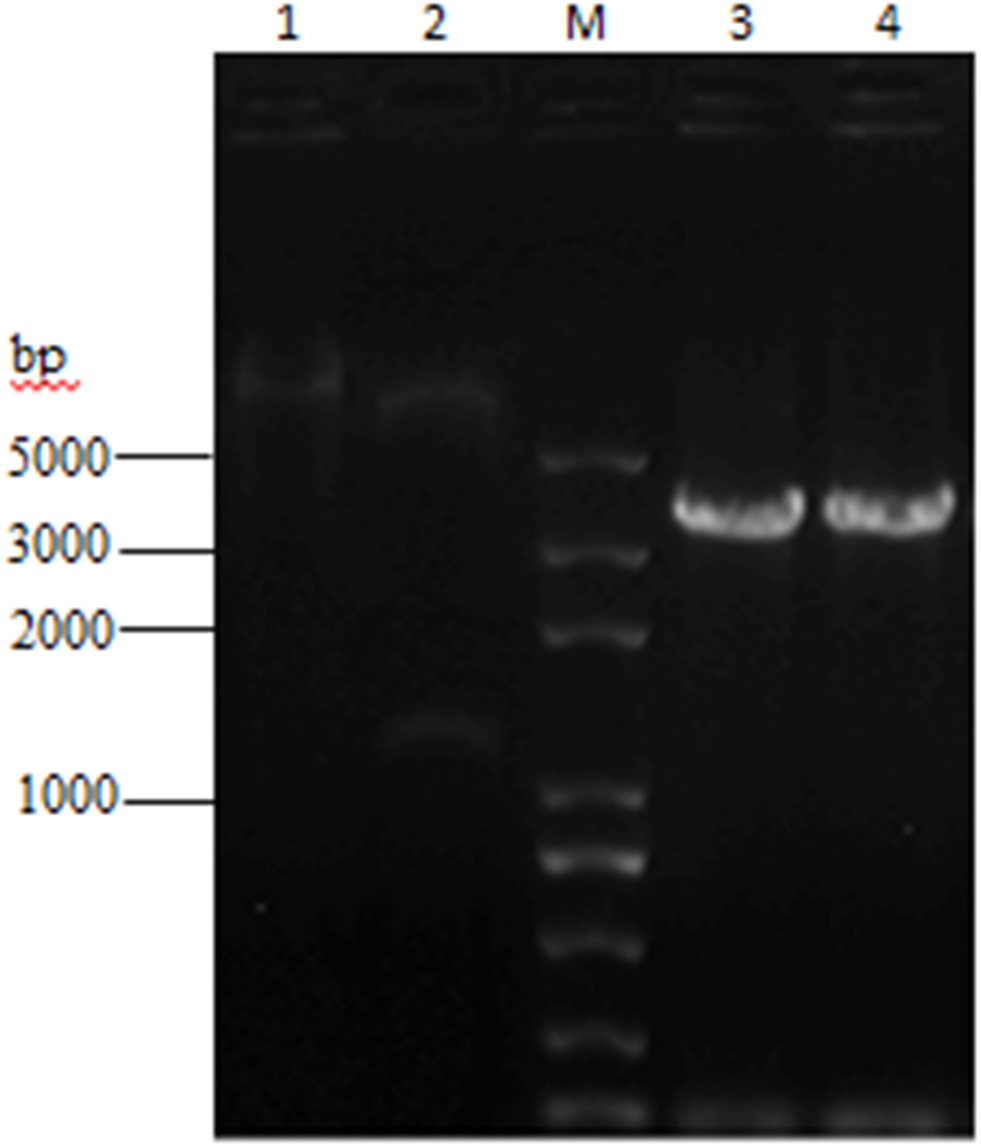
Agarose gel electrophoresis analysis of 3 combinant expression plasmid. The plasmid was confirmed by PCR and double digestion using NcoI and XhoI. Lane 1 was the fragment of recombinant plasmid DNA pTriEx-1.1 Hygro-β_2_-AR. Lane 2 was the electrophoresis results of digested products containing 2 fragments (6951 bp and 1257 bp). A 3300 bp fragment (lane 3 and lane 4) was amplified by PCR from the recombinant plasmid, which was identical with the sum of the size of target gene and vector sequences between NcoI and XhoI.

**Fig 2:**
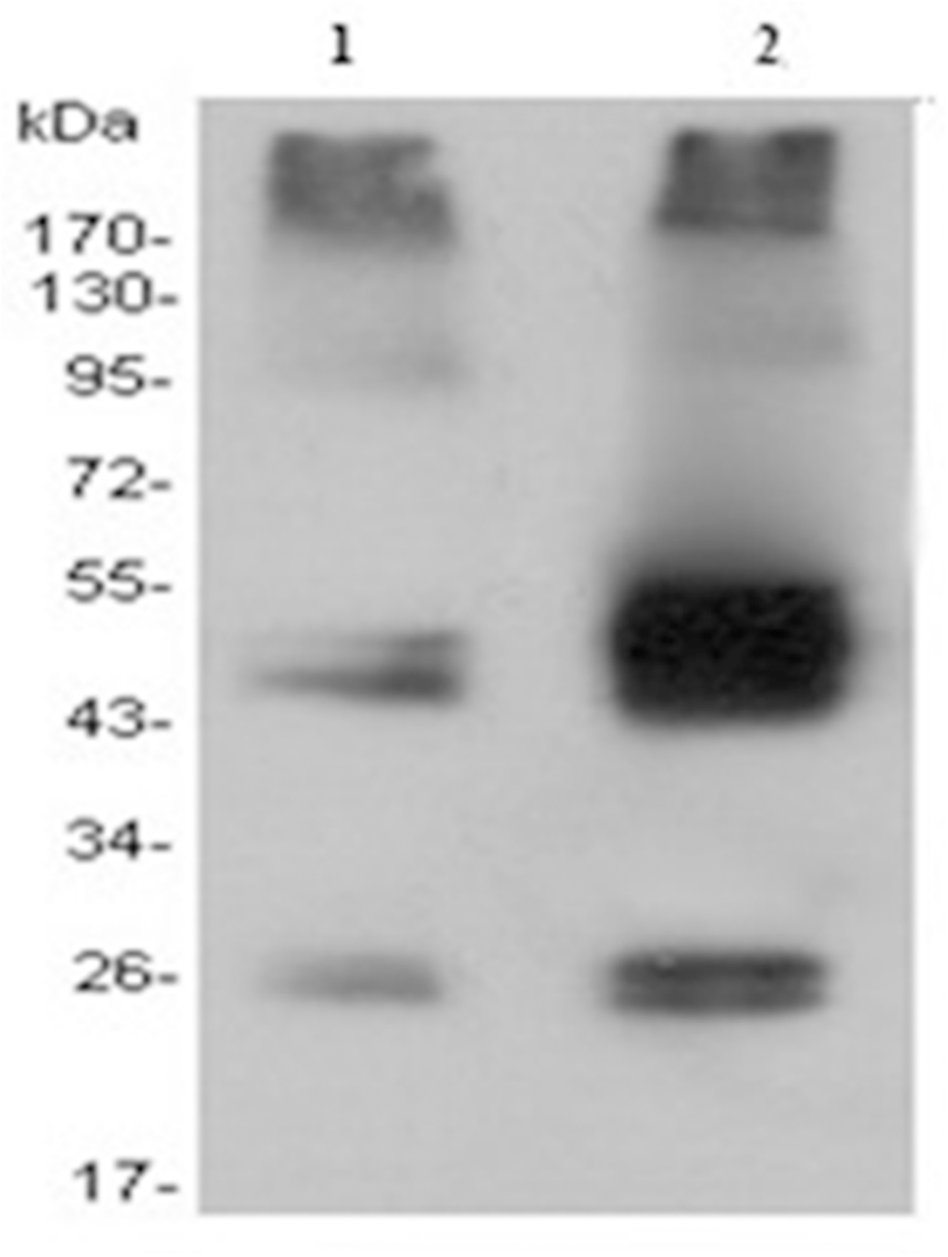
Western blot analysis of membrane protein extracts isolated from instantaneous transfected and nontransfected HEK293 cells. Equal amounts of proteins were loaded for each of the strains, separated by gel electrophoresis, and Western blotted with anti-His monoclonal antibodies. Membrane protein extracts of nontransfected (lane 1) and instantaneous transfected (lane 2) HEK293 cells, (lane M) molecular weight standards. The receptor produced in HEK293 cells appeared in cellulose acetate membrane as diffuse bands with an apparent molecular mass between 43 and 55 kDa.

Recombinant plasmid of pTriEx-1.1 Hygro-β_2_-AR was constructed successfully after verification of PCR (Fig 1, lanes 3-4), double enzyme digestion (Fig 1, lanes 1-2), and sequencing. As the plamid could be used to test the expression in 3 systems of *E*. *coli*, insect, and mammalian cells, the expression study was performed first in *E*. *coli* BL21 (DE3); however, the expression level indicated by SDS-PAGE and ELISA was very low (data not shown). Consequently, transient expression of β_2_-AR gene in HEK293 cells was explored and the approximate densities ranged from 17 to 23 pmol/mg of membrane protein. This expression level was still not completely satisfactory, but could basically meet the requirement of its exploration in the development of a new detection method. In order to obtain higher amount of the recombinant receptor, site-specific mutagenesis of β_2-_AR gene or optimization of expression vector would be useful for futher reseach. Western blot was probed with anti-His tag monoclonal antibody, and the results revealed that β_2_-AR expressed in HEK293 cells appeared in electrophoresis as diffuse bands with an apparent molecular mass in the range of 47-55 kDa (Fig 2, lane 1), compared with the cellular proteins of nontransfected HEK293 cells (Fig 2, lane 2). The molecular mass range of the target protein was close to the molecular mass (46.73 kDa) calculated from the amino acid sequence of monomeric β_2_-AR. One of the interpretation of the result was that glycosylation contributed to 4 and 8 kDa mass increment [27]. To reach the highest expression level of recombinant β_2_-AR, the optimal expression time was determined as 72 hours after transfection (data not shown).

**Fig 3:**
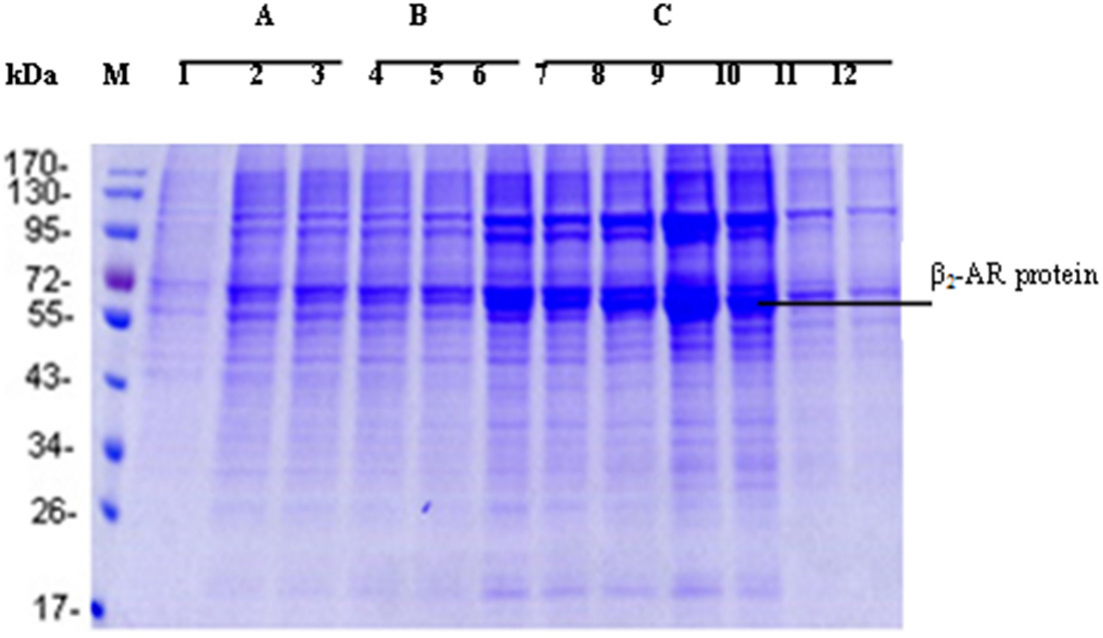
SDS-PAGE of purified membrane protein by Ni-affinity chromatography in different imidazole concentration. The imidazole concentration in the elution buffer was optimized at 3 levels: 75 (A), 125 (B), and 250 Mm (C). When it reached 250 mM, the eluate in 7-10 tubes contained abundant recombinant β_2_-AR protein.

**Fig 4:**
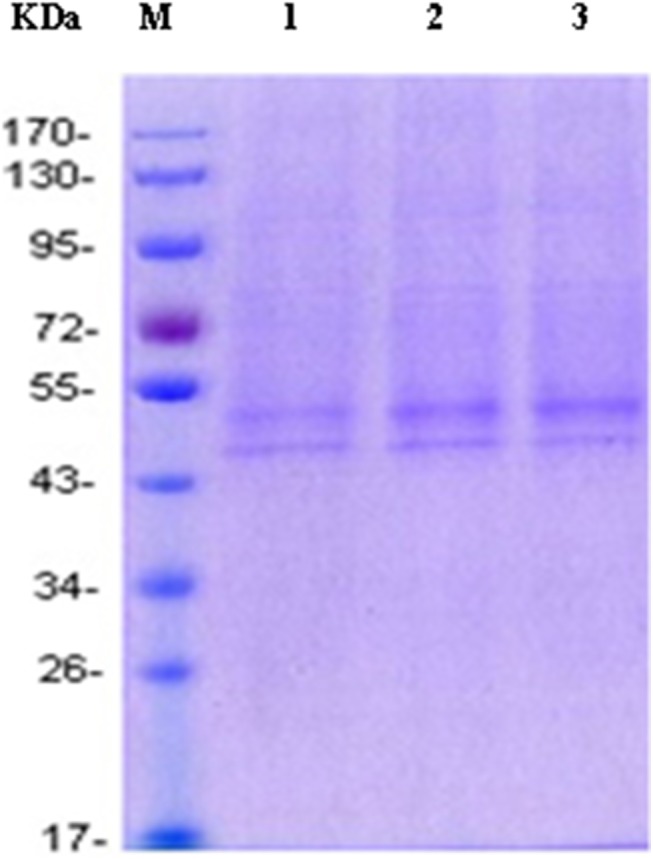
SDS-PAGE of the purified β_2_-AR protein from cells transfected with the plasmid of pTriEx-1.1 Hygro-β_2_-AR. (Lanes 1-3) 10 μL of Ni-NTA-purified protein, (lane M) molecular weight standards. The receptor expressed in HEK293 was glycosylated and migrated as 2 bands, with the minor band showing an apparent molecular mass of around 47 kDa and the major band around 52 kDa.

Based on the principle of combination between His-tag protein and Ni^2+^, the recombinant receptor was purified by Ni-affinity chromatography adopted with Ni-NTA produced by Qiagen. Unlike Ni-iminodiacetic acid chelated by Ni^2+^ with triple bond, the purification filler was chelated by Ni^2+^ with quadrivalent bond free from abscission in the maximal degree, so as to guarantee the protein of high purity. To realize the best eluting effect, the imidazole concentration in the elution buffer was optimized. In Fig 3, it can be observed that β_2_-AR was eluted completely when the imidazole concentration reached 250 mmol/L in the elution buffer. The purified β_2_-AR appeared in electrophoresis as 2 predominant forms of 52 kDa and 47 kDa, respectively (Fig 4). It was stored at -80°C at a concentration of 80,000 μg/L.

### Activity assay and optimization of β_2_-AR-based ELRA

HRP-β-agonists were used in this study as binding ligands to establish a convenient and safe activity assay. As can be observed from Table 1 and Table A in S1 Document, the recombinant receptor could bind all 3 HRP-β-agonists, and ranking based on their binding affinity was HRP-CBL>HRP-SAL>HRP-RAC. Thus, HRP-CBL was chosen as the enzyme marker to the development of ELRA, and its optimal working concentration in ELRA was 1:1000 dilution, as the OD_450_ value at this concentration was approximately 1.0.

**Table 1:** Activity assay of recombinant β_2_-AR.

Dilution rate	HRP-clenbuterol (OD_450_)	HRP-ractopamine (OD_450_)	HRP-salbutamol (OD_450_)
1:500	1.287	0.644	0.782
1:1000	1.024	0.439	0.531
1:3000	0.521	0.277	0.423
1:6000	0.369	0.113	0.155

The performance of ELRA was always influenced by some parameters, so the system was further optimized according to the OD_450_ value including coating buffer, blocking buffer, and blocking process. Table 2, Tables B and C in S1 Document illustrated that carbonate-buffered solution (0.05 M, pH 9.6) exhibited better coating effect than PBS (0.02 M, pH 7.2) and Tris-HCl (0.05 M, pH 7.5), and 1% BSA was the most suitable blocking buffer among the 3 candidates. The data were compared after the blocked plates were placed in 4°C overnight and 37°C for 2 hours. The results of the former (OD_450_ = 1.097) was significantly better than that of the latter (OD_450_ = 0.672). A possible explanation for this might be that higher temperature might lead to decreased β_2_-AR activity.

**Table 2:** Optimization of the coating buffer and blocking buffer.

Coating buffer (OD_450_)		Blocking buffer (OD_450_)	
**CBS**	1.109±0.010	**1% BSA**	1.014±0.012
**PBS**	0.927±0.011	**5% skim milk**	0.921±0.010
**Tris-HCl**	1.015±0.008	**1% OVA**	0.968±0.009

### Establishment of calibration curve

**Fig 5:**
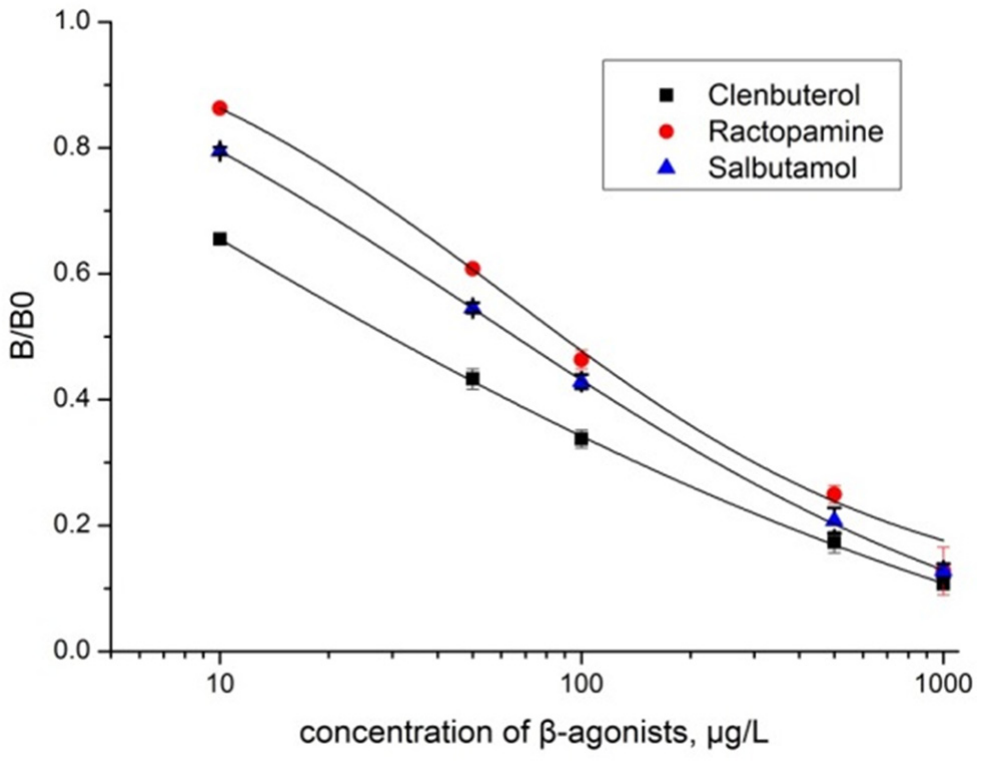
The calibration curves constructed by plotting the B/B0 ratio against the concentration of β-agonists. Each value represents the mean ± SD from 3 independent measurements.

A typical calibration curve was constructed and illustrated in Fig 5 by plotting [B/B0] × 100% against the β-agonist concentration, where B and B0 were the absorbance of the β-agonist at the standard point and at zero concentration of the β-agonist, respectively (Table D in S1 Document). The IC50 values of CBL, SAL, and RAC calculated from the fitting equations were 34, 53, and 63 μg/L, respectively. The IC50 values in this study were much lower than that of the reported radiolabeled receptor assay (higher than 110 μg/L) [37], and in the same order of magnitude with the previous ELRA method [38]. Besides, among the above 3 studies, the lowest IC50 value of each study originated from CBL, SAL, and RAC, respectively. The difference may be ascribed to several possible reasons. However, the main reason was the different structure and binding affinity caused by the various sources of β_2_-AR. Moreover, different expressions and detection systems might also contribute to it.

### Cross-reactivity and detection limit

In theory, β_2_-AR has broad-spectrum binding affinities with β-agonist matter, but the binding affinities were changed along with the difference of structures. Both RAC and SAL exhibited high cross-reactivity to CBL, with values of 54.0% and 64.2%, respectively. Besides, the cross reactivity of RAC with SAL was 84.1%. These findings achieved the expected purpose of multiresidue determination. The LOD was evaluated by analyzing 20 randomly selected swine urine samples that were free of β-agonist. The LOD calculated as the mean + 3 SD was 4 μg/L.

### Detection of β-agonist in swine urine samples

The recovery studies were conducted to evaluate the accuracy and precision of the β_2-_AR-based ELRA system. The urine samples were respectively spiked with 1, 10, 50, and 100 μg/L of each β-agonist after centrifugation at 10,000 rpm for 30 minutes, after which the recovery was analyzed by the assay.

The average recovery rates of CBL, RAC, and SAL were 68.2% (varying from 52.2% to 82.4%), 60.3% (varying from 43.8% to 71.2%), and 65.5% (varying from 48.7% to 75.1%), respectively (Table 3 and Table E in S1 Document), which were lower than those from the previous report [37]. It was probably because of the greater matrix effect of urine compared with the feedstuffs adopted in that research. This inference suggests that more sophisticated pretreatment process may help enhancement of recovery. All CVs were within 15%, indicating an ideal variation extent.

**Table 3:** Recovery of β–agonist sdetermined by developed ELRA.β-agonist.

	Spikedconcentration (ng/mL)	Measured concentration (ng/mL)	Recovery (%)	Coefficient of variation (CV) (%)
**clenbuterol**	1	0.52±0.06	52.0	11.5
	10	6.38±0.67	63.8	10.5
	50	37.30±3.23	74.6	8.7
	100	77.10±7.08	77.1	9.2
**ractopamine**	1	0.43±0.04	43.0	9.3
	10	5.64±0.83	56.4	14.7
	50	34.50±4.00	69.0	11.6
	100	71.20±8.66	71.2	12.2
**salbutamol**	1	0.48±0.07	48.0	14.6
	10	6.53±0.75	65.3	11.5
	50	36.20±3.50	72.4	9.7
	100	75.70±7.13	75.7	9.4

Results are mean ± SD (n = 5).

Recovery (%) = (detected concentration/spiked concentration) ×100.

CV (%) = (S.D./mean) × 100%.

All β-agonists must not be found in the sample in accordance with relevant Chinese laws. The sensitivity of the commercial ELISA kits for β-agonists were in the range of 0.025–0.1 μg/L, and most of their recoveries were between 70%-110%. In contrast, the results obtained in this study still had a certain gap with them. However, the method showed the capability of the receptor to bind structurally different β-agonists which couldn't be matched by any current rapid detection method. Therefore, with the improvement, this assay would be a valuble tool to be applied for the screening of β-agonists residues in animal production.

## Conclusions

A novel receptor assay based on recombinant β_2_-AR expressed in HEK293 cells was developed for simultaneous detection of 3 β-agonists, including CBL, RAC, and SAL. The method had a lower limit detection of 4 μg/L. Although the average recovery rates obtained for the 3 β-agonists (60.3%-68.2%) was unsatisfactory, it revealed the potential of the method to be used to detect known or unknown compounds with agonistic activity in different matrix. To achieve this goal, many aspects should be further investigated such as the increased activity of recombinant receptor as well as the optimization of reaction system and sample pretreatment.

In conclusion, this approach is simple, quick, and environment friendly, showing a potential application for rapid screening of a group of β-agonists for the control of the illegal abuse of β-agonists in animal production.

## Supporting Information

S1 Document Supporting information document. This document contains five Tables (A. Original date of activity assay of recombinant β_2_-AR, B. Original date of optimization of the coating buffer, C. Original date of optimization of blocking buffer, D. Original date of establishment of calibration curve, and E. Original date of recovery of β–agonist sdetermined by developed ELRA).
(DOCX)
