# Supplementary material for: Multiresidue Method for Analysis of β Agonists in Swine Urine by Enzyme Linked Receptor Assay Based on β2 Adrenergic Receptor Expressed in HEK293 Cells
Source: PLoS One. 2015 Sep 30;10(9):e0139176. doi: 10.1371/journal.pone.0139176 (PMC4589316; doi:10.1371/journal.pone.0139176)
Supplement: S1 Document — This document contains five Tables (A. Original date of activity assay of recombinant β2-AR, B. Original date of optimization of the coating buffer, C. Original date of optimization of blocking buffer, D. Original date of establishment of calibration curve, and E. Original date of recovery of β–agonist sdetermined by developed ELRA). (DOCX) [file pone.0139176.s001.docx]

**Supporting Information**

**Table A Original date of activity assay of recombinant β_2_-AR**

| **Dilution rate** | | **HRP-clenbuterol** | **HRP-ractopamine** | **HRP-salbutamol** |
| --- | --- | --- | --- | --- |
| **1:500** | average | 1.286666667 | 0.644333333 | 0.781666667 |
|  | 1 | 1.293 | 0.645 | 0.788 |
|  | 2 | 1.279 | 0.651 | 0.776 |
|  | 3 | 1.288 | 0.637 | 0.781 |
| **1:1000** | average | 1.024333333 | 0.438666667 | 0.530666667 |
|  | 1 | 1.027 | 0.432 | 0.528 |
|  | 2 | 1.031 | 0.448 | 0.541 |
|  | 3 | 1.015 | 0.436 | 0.523 |
| **1:3000** | average | 0.521333333 | 0.277 | 0.423333333 |
|  | 1 | 0.526 | 0.283 | 0.438 |
|  | 2 | 0.529 | 0.291 | 0.411 |
|  | 3 | 0.509 | 0.257 | 0.421 |
| **1:6000** | average | 0.368666667 | 0.113333333 | 0.155333333 |
|  | 1 | 0.362 | 0.119 | 0.161 |
|  | 2 | 0.379 | 0.107 | 0.157 |
|  | 3 | 0.365 | 0.114 | 0.148 |

**Table B Original date of optimization of the coating buffer**

|  | **OD_450_** | **OD_450_** | **OD_450_** | **average** | **SD** |
| --- | --- | --- | --- | --- | --- |
| **CBS** | 1.098 | 1.116 | 1.114 | 1.109333 | 0.0099865766 |
| **PBS** | 0.936 | 0.931 | 0.915 | 0.927333 | 0.010969655 |
| **Tris-HCl** | 1.021 | 1.006 | 1.017 | 1.014667 | 0.007767453 |

**Table C** **Original date of optimization of blocking buffer**

|  | **OD_450_** | **OD_450_** | **OD_450_** | **average** | **SD** |
| --- | --- | --- | --- | --- | --- |
| **1% BSA** | 1.027 | 1.009 | 1.005 | 1.013667 | 0.01171893 |
| **5% skim milk** | 0.919 | 0.912 | 0.931 | 0.920667 | 0.00960902 |
| **1% OVA** | 0.959 | 0.977 | 0.969 | 0.968333 | 0.0090185 |

**Table D Original date of establishment of calibration curve**

|  |  | **concentration of β‑agonists (μg/L)** | | | | | |
| --- | --- | --- | --- | --- | --- | --- | --- |
|  |  | 0 | 10 | 50 | 100 | 500 | 1000 |
| **CBL** | average | 0.967 | 0.633333 | 0.418667 | 0.326667 | 0.168 | 0.104 |
|  | 1 | 0.971 | 0.638 | 0.411 | 0.309 | 0.178 | 0.117 |
|  | 2 | 0.958 | 0.635 | 0.437 | 0.333 | 0.181 | 0.098 |
|  | 3 | 0.972 | 0.627 | 0.408 | 0.338 | 0.145 | 0.097 |
|  | SD | 0.006377 | 0.004643 | 0.013021 | 0.012658 | 0.01631 | 0.009201 |
| **RAC** | average | 0.959 | 0.827667 | 0.583 | 0.444667 | 0.239333 | 0.122667 |
|  | 1 | 0.966 | 0.838 | 0.592 | 0.472 | 0.253 | 0.144 |
|  | 2 | 0.948 | 0.819 | 0.577 | 0.431 | 0.218 | 0.153 |
|  | 3 | 0.963 | 0.826 | 0.58 | 0.431 | 0.247 | 0.071 |
|  | SD | 0.007874 | 0.007846 | 0.006481 | 0.019328 | 0.015283 | 0.036718 |
| **SAL** | average | 0.951 | 0.756 | 0.519 | 0.407667 | 0.197667 | 0.122 |
|  | 1 | 0.955 | 0.783 | 0.533 | 0.419 | 0.205 | 0.134 |
|  | 2 | 0.961 | 0.779 | 0.529 | 0.389 | 0.211 | 0.141 |
|  | 3 | 0.937 | 0.706 | 0.495 | 0.415 | 0.177 | 0.091 |
|  | SD | 0.010198 | 0.035393 | 0.017049 | 0.0133 | 0.014817 | 0.022106 |

**Table E Original date of recovery of β–agonist sdetermined by developed ELRA**

| **CBL** | **concentration of β‑agonists (μg/L)** | **1** | **10** | **50** | **100** |
| --- | --- | --- | --- | --- | --- |
|  | **average** | 0.5202 | 6.3798 | 37.2996 | 77.10 |
|  | **1** | 0.578 | 5.614 | 36.302 | 77.562 |
|  | **2** | 0.445 | 5.716 | 42.283 | 84.133 |
|  | **3** | 0.496 | 6.688 | 37.426 | 66.757 |
|  | **4** | 0.496 | 7.090 | 37.171 | 82.984 |
|  | **5** | 0.586 | 6.791 | 33.316 | 74.064 |
|  | **SD** | 0.06020133 | 0.669988 | 3.229979 | 7.079776 |
| **RAC** | **concentration of β‑agonists (μg/L)** | **1** | **10** | **50** | **100** |
|  | **average** | 0.43 | 5.6396 | 34.4996 | 71.2004 |
|  | **1** | 0.477 | 5.606 | 29.408 | 80.513 |
|  | **2** | 0.402 | 6.729 | 31.086 | 74.739 |
|  | **3** | 0.462 | 5.182 | 37.370 | 76.566 |
|  | **4** | 0.429 | 4.573 | 38.391 | 60.360 |
|  | **5** | 0.380 | 6.108 | 36.243 | 63.824 |
|  | **SD** | 0.04036707 | 0.829812 | 3.999959 | 8.659697 |
| **SAL** | **concentration of β‑agonists (μg/L)** | **1** | **10** | **50** | **100** |
|  | **average** | 0.4802 | 6.5304 | 36.20 | 75.70 |
|  | **1** | 0.545 | 6.558 | 37.828 | 80.549 |
|  | **2** | 0.526 | 6.988 | 36.900 | 70.246 |
|  | **3** | 0.366 | 6.208 | 36.087 | 66.730 |
|  | **4** | 0.474 | 5.469 | 30.435 | 83.936 |
|  | **5** | 0.490 | 7.429 | 39.751 | 77.040 |
|  | **SD** | 0.06977249 | 0.749692 | 3.499928 | 7.129991 |
